# Untargeted analysis in post-COVID-19 patients reveals dysregulated lipid pathways two years after recovery

**DOI:** 10.3389/fmolb.2023.1100486

**Published:** 2023-03-03

**Authors:** Yamilé López-Hernández, Juan José Oropeza-Valdez, David Alejandro García Lopez, Juan Carlos Borrego, Michel Murgu, Jorge Valdez, Jesús Adrián López, Joel Monárrez-Espino

**Affiliations:** ^1^ CONACyT-Metabolomics and Proteomics Laboratory, Academic Unit of Biological Sciences, Autonomous University of Zacatecas, Zacatecas, Mexico; ^2^ Metabolomics and Proteomics Laboratory, Academic Unit of Biological Sciences, Autonomous University of Zacatecas, Zacatecas, Mexico; ^3^ Doctorado en Ciencias Básicas, Universidad Autónoma de Zacatecas, Zacatecas, Mexico; ^4^ Departamento de Epidemiología, Hospital General de Zona #1 “Emilio Varela Luján”, Instituto Mexicano del Seguro Social, Centro, Zacatecas, Mexico; ^5^ Waters Technologies of Brazil, Alameda Tocantins, Barueri, Brazil; ^6^ Waters Corporation, Mexico City, Mexico; ^7^ MicroRNAs and Cancer Laboratory, Academic Unit of Biological Sciences, Autonomous University of Zacatecas, Zacatecas, Mexico; ^8^ Department of Health Research, Christus Muguerza del Parque Hospital Chihuahua, University of Monterrey, San Pedro Garza García, Mexico

**Keywords:** long covid, metabolomics, metabolite, lipidomics, post-COVID-19

## Abstract

**Introduction:** Similar to what it has been reported with preceding viral epidemics (such as MERS, SARS, or influenza), SARS-CoV-2 infection is also affecting the human immunometabolism with long-term consequences. Even with underreporting, an accumulated of almost 650 million people have been infected and 620 million recovered since the start of the pandemic; therefore, the impact of these long-term consequences in the world population could be significant. Recently, the World Health Organization recognized the post-COVID syndrome as a new entity, and guidelines are being established to manage and treat this new condition. However, there is still uncertainty about the molecular mechanisms behind the large number of symptoms reported worldwide.

**Aims and Methods:** In this study we aimed to evaluate the clinical and lipidomic profiles (using non-targeted lipidomics) of recovered patients who had a mild and severe COVID-19 infection (acute phase, first epidemic wave); the assessment was made two years after the initial infection.

**Results:** Fatigue (59%) and musculoskeletal (50%) symptoms as the most relevant and persistent. Functional analyses revealed that sterols, bile acids, isoprenoids, and fatty esters were the predicted metabolic pathways affected in both COVID-19 and post-COVID-19 patients. Principal Component Analysis showed differences between study groups. Several species of phosphatidylcholines and sphingomyelins were identified and expressed in higher levels in post-COVID-19 patients compared to controls. The paired analysis (comparing patients with an active infection and 2 years after recovery) show 170 dysregulated features. The relationship of such metabolic dysregulations with the clinical symptoms, point to the importance of developing diagnostic and therapeuthic markers based on cell signaling pathways.

## 1 Introduction

Post-COVID-19 exists, and matters. The World Health Organization (WHO) has recently recognized the post-COVID-19 (or long COVID) condition (Delphi consensus), as one “that occurs in individuals with a previous history of probable or confirmed SARS-CoV-2 infection, usually three months after the onset, with symptoms lasting at least two months that cannot be explained by an alternative diagnosis” (Soriano et al., 2022). Studies show that 60% of COVID-19 survivors experience post-COVID symptoms (Fernández-de-Las-Peñas et al., 2021), and that these are associated with worse quality of life (Malik et al., 2022).

Why some patients experience long-term symptoms after COVID-19 infection remains uncertain (Yong, 2021). Genetic susceptibility, age, and viral load could be related to this syndrome (Collaborators, 2022). Organ damage due to an excessive inflammatory response caused by the virus, persistent reservoirs of SARS-CoV-2 in certain tissues triggering post-infection morbidity, pathogen reactivation as a result of immune dysregulation, host-microbiome alterations, coagulation problems, and autoimmunity due to molecular mimicry between SARS-CoV-2 and autoantibodies could be involved mechanisms (Collaborators, 2022). In addition, it has also been suggested that the prolonged symptoms of COVID-19 may not be a direct result of SARS-CoV-2 infection, but rather the consequence of Epstein-Barr virus reactivation induced by COVID-19 inflammation (Ortona and Malorni, 2022).

Multiple studies point to an immunometabolic dysregulation, highlighting certain metabolites, such as those involved in the tryptophan and kynurenine pathways, which have a very important role in the immune system. To date, the plethora of symptoms and protracted disorders documented suggest that various concurrent mechanisms might be involved, and that different therapeutic approaches need to be established when dealing with these patients.

It remains unknown whether SARS-CoV-2 can cause substantial tissue damage leading to a chronic form of the disease, such as the chronic convalescent lesions seen with other viral infections including the Human Immunodeficiency Virus (HIV), hepatitis C virus (HCV), hepatitis B virus (HBV) and some herpesviruses. Previous studies with SARS survivors have shown lung abnormalities months after infection (Lopez-Leon et al., 2021).

A recent comprehensive molecular investigation revealed extensive inflammation and degeneration in the brains of patients who died of COVID-19, even among those with no reported neurological symptoms. In that study, the authors report that SARS-CoV-2 virus induced vascular damage affecting endothelial cells and caused generalized neuroinflammation. Cytokines like interleukin-1 and interleukin-6 were highly elevated in patients with COVID-19, which are the ones driving neurodegeneration and Alzheimer’s disease (Reiken et al., 2022).

Another study showed that between one and 12 months after infection, patients recovered from COVID-19 are at increased risk of incident cardiovascular disease, including cerebrovascular disorders, arrhythmias, ischemic and non-ischemic heart disease, pericarditis, myocarditis, heart failure, and thromboembolic events; these risks were documented even among people who were not hospitalized during the acute phase of the infection, and increased gradually depending on the care setting during the acute phase (non-hospitalized, hospitalized, and intensive care) (Xie et al., 2022).

In terms of mortality, a large study comprising nearly five million healthy controls and 90,000 COVID-19 patients revealed that the risk of death among COVID-19 survivors in the following 6 months after infection, increased by 60% (Al-Aly et al., 2021).

In the present work, we investigated the health status of patients who recovered from a mild, severe, and critical COVID-19 in 2020 (post-COVID-19 patients). Two years later, patients who provided informed consent were surveyed. Clinical symptomatology and blood samples for laboratory analyses were obtained. Untargeted lipidomic analysis was performed with plasma samples to assess potential dysregulation of lipid metabolism. These long-term alterations need to be deeply analyzed to find a possible connection with symptoms persistence and to find effective therapeutic alternatives to treat (or cure) these patients.

## 2 Materials and methods

### 2.1 Patients’ recruitment

Symptomatic individuals aged 35–70 years who were RT-qPCR-tested for SARS-CoV-2 between March 15 and 1 November 2020, at the Zacatecas General Hospital’s Respiratory Triage Unit of the Mexican Institute of Social Security (IMSS) and Christus Muguerza del Parque Hospital of Chihuahua city were included in this study. Negative controls were RT-qPCR negative patients. Inclusion criteria for negative controls, and mild, severe, and critically ill patients are shown in Supplementary Table S1.

The COVID-19 group were patients who were positive for SARS-CoV-2 in the first epidemic wave (2020). Blood specimens for plasma isolation were collected within two days after hospital admission on average. Baseline information including age, sex, comorbidities, clinical and laboratory data, and disease severity classification according to WHO guidelines (WHO, 2023).

Clinical data was obtained from the electronic medical records of each patient and stored by a password-protected database and provided in Table 1. Blood samples were collected in 2020 and stored at −80°C in the biobank at the Autonomous University of Zacatecas, Mexico.

**TABLE 1:** Clinical and demographic characteristics.

Variable	Negative controls (n = 15)	COVID-19 (n = 28)	Post-COVID-19 (n = 20)	*p*-value
Age, mean ± SD (years)	47.2 ± 8.4	56.3 ± 13	51.8 ± 11.6	0.0537
Male gender, n (%)	9 (60)	15 (53.5)	11 (55)	0.9198
Smoking, n (%)	3 (20)	2 (7.6)	3 (15)	0.4501
**Comorbidities (self-reported), n (%)**				
Diabetes	1 (6.66)	10 (35.7)	3 (15)	0.0592
Hypertension	4 (26.6)	8 (28.5)	9 (45)	0.4044
Obesity	1 (6.6)	8 (28.5)	0 (0)	**0.0128** ^*b^
**Symptomatology, n (%)**				
Fever	0 (0)	15 (53.5)	0 (0)	<0.0001 * ^**a, b**^
Cough	0 (0)	24 (85.7)	3 (15)	**<0.0001** ^*a, b^
Headache	10 (66)	18 (64)	6 (3)	**0.0334** ^*b, c^
Dyspnea	2 (13.3)	23 (82.1)	6 (3)	**<0.0001** ^*a, b^
Diarrhea	0 (0)	6 (21.4)	2 (10)	0.1201
Chest tightness	0 (0)	14 (50)	4 (20)	**0.0015** ^*a, b^
Pharyngalgia	8 (53.3)	9 (32.1)	2 (10)	**0.0209** ^*c^
Myalgia	8 (53.3)	18 (64.2)	9 (45)	0.4072
Arthralgias	5 (33.3)	18 (64.2)	10 (50)	0.1482
Anosmya	0 (0)	5 of 14 (35)	1 (5)	**0.006** ^*a, b^
**Laboratory data, median (Q1-Q3)**				
Erythrocytes (million/mL)	5.3 (5–5.5)	4.8 (1.2–5.32)	5 (0.9–5.48)	0.0935
Hemoglobin (g/dL)	15.5 (14.9–16.4)	13.25 (9.78–15.2)	15.45 (14.63–16.55)	**0.001** ^*a, b^
Platelets (thousands/mL)	277 (242–320)	233.5 (145.5–306.5)	237 (216–248)	0.1183
Leukocytes (×10^3^)	6.6 (5.9–7.8)	8.3 (5.08–11.68)	7.23 (6.38–7.98)	0.4093
Lymphocytes (%)	33.3 (25.8–37)	7 (2.25–12.8)	34.95 (29.88–38.25)	**<0.0001** ^*a, b^
Monocytes (%)	6.5 (5.4–8.3)	2.95 (0.4–4.78)	6.3 (1.16–7.1)	**0.0036** ^*a^
Neutrophils (%)	58 (51.3–62.7)	81.45 (26.56–91.88)	51.1 (8.82–58.1)	**<0.0001** ^*a, b^
Glucose (mg/dL)	91 (84–116)	123 (80–191.3)	100.3 (19.02–131.9)	0.1314
Creatinine (mg/dL)	1 (0.7–1.1)	0.67 (0.5–0.78)	0.795 (0.7–0.92)	**0.0083** ^*a^

*a: Negative Controls vs. COVID-19.

*b: COVID-19, vs. post-COVID-19.

*c: Negative Controls vs. post-COVID-19.

Significant values (*p* < 0.05) are highlighted in bold.

Two years after hospital discharge and recovery, plasma samples were obtained from 22 COVID-19 patients (post-COVID-19 group). Chest computed tomography (CT) scans (in patients that had a baseline CT), basic blood biochemical markers (i.e., hemoglobin, platelets, leukocytes, lymphocytes, and creatinine), and a questionnaire to assess the persistence of clinical symptoms were used to evaluate their clinical recovery. Blood collection was done in fasting conditions. Only three patients had one reinfection 20 months on average after recovery from the initial infection.

The study was conducted in accordance with the Declaration of Helsinki (1976). It was also revised and approved by the Research and Ethics Committees of the Instituto Mexicano de Seguridad Social, with the registration number R-2022-3301-038 and Christus Muguerza del Parque Hospital (folio HCMP-CEI-28022022-A01 and HCMP-CEI-15042020-3). Informed consent was obtained from all participants. All patients included in this study were informed in writing regarding the collection of their samples for research aims and were given the right to refuse participation.

### 2.2 Sample preparation

Blood collected in vacutainer tubes (EDTA) was centrifuged at 4°C and 3000 g for 15 min. Plasma was aliquoted and stored at −80°C until use. Plasma thawed in ice was extracted with pre-cooled isopropanol in a 1:3 ratio (LCMS grade, Honeywell, Charlotte, NC, United States) (Medina et al., 2020) vortexed for 1 min and incubated at −20°C overnight for protein precipitation. Subsequently, the extraction mix was centrifuged at 16000 *g* and 4°C for 15 min and supernatants were carefully collected. An additional step of centrifugation was done to remove any debris collected. For the analysis, each aliquot was transferred into certified LC vials (TruView LCMS, United States) and diluted to 1:20 ratio with a mixture of isopropanol/acetonitrile/water (2:1:1, v: v: v). Sample preparation order was randomized for sample picking to ensure no systematic biases were present during sample preparation.

### 2.3 Quality controls (QC) and quality assurance (QA)

These processes are referred to as the procedures applied in preparation for data acquisition (QA) and during/after data acquisition (QC) (Kirwan et al., 2022). As part of QA procedures, the equipment was subjected to a complete maintenance twice a year. This maintenance included both the chromatography system, the mass analyzer, and the nitrogen source. Sample cone and ion source cleaning were performed between every analytical batch. Calibration and manual tuning were also performed immediately before running samples. Temperature control, standardized protocols of operations and qualification of technical staff were also considered.

A pool of human plasma from all participants in the study served as a technical replicate throughout the dataset (pooled QCs). QCs were prepared identically as individual samples. Overall process variability was determined by calculating the median relative standard deviation (RSD) for all endogenous metabolites present in 100% of the pooled QCs samples. Experimental samples were randomized across the platform run with 10 QCs samples at the beginning (for instrument and column equilibration) and one QC sample was acquired every ten samples injected.

### 2.4 Ultra-performance liquid chromatography (UPLC)-Mass spectrometry method for lipidomic analysis

The analysis was performed using an ACQUITY UPLC I-Class (Waters Corp., Milford, MA, United States) coupled to a XEVO-G2 XS quadrupole time-of-flight (ToF) mass spectrometer (Waters, Manchester, NH, United States) with an electrospray ionization source. The samples were analyzed in positive (ESI+) mode.

A UPLC CSH C18 column (2.1 × 100 mm, 1.7 µm) with a binary gradient elution of solvents was used for lipid separation. The mobile phase A was 10 mM ammonium formate with 0.1% formic acid in acetonitrile/water (60:40, v:v) and mobile phase B was 10 mM ammonium formate with 0.1% formic acid in isopropanol/acetonitrile (90:10, v:v). The mobile phases were delivered at a flow rate of 0.3 mL/min, initially with 60% A, followed by a linear gradient to 57% A over 2 min, and then the percentage of A was decreased to 50% within 0.5 min. Over the next 10 min, the gradient was ramped to 46% A, and the amount of A was then decreased to 30% in 0.5 min. Over 6 min, the amount of A decreased to 1%, and returned to initial conditions (60%) at the end of 25 min. The column temperature was adjusted to 55°C and the injection volume was five uL. Data was acquired using positive electrospray ionization mode with the capillary voltage set to 3.2 kV, the cone voltage to 40 eV and the source temperature to 130 °C. The desolvation gas was nitrogen, with a flow rate of 900 L/h, cone gas flow of 25 L/h and temperature of 550°C. Data was acquired in the m/z range of 50–1,200 in data independent analysis (DIA) mode in which the collision energy was alternated between low energy (6 eV) and high energy (ramped from 10–40 eV) in consecutive scans of 0.2 s generating high and low chromatograms and spectra. Lockmass correction was made by the acquisition of mass reference leucine enkephalin in intervals of 30 s.

#### 2.4.1 Data analysis

Raw data were processed under default parameters as a UNIFI file (UNIFI 1.8.2, Waters Corp., Milford, United States), which was exported to Progenesis QI (version 3.0.7, Waters Corp., Milford, United States). For the alignment, retention times below 0.5 min and after 18 min were excluded. A width peak of 0.06 s was defined. Deconvolution was automatically performed, considering M + H, M + Na, M + H-H20, M + K, and M + NH4 as adducts. However, manual inspection was done, eliminating those features with incorrect alignment in chromatograms and neutral and m/z mass. An excel file was exported and a signal to noise (S/N) ratio was calculated for each sample based on the extraction blank. All features with a S/N < 5 in the 80% of samples were eliminated. Besides, RSD was calculated taking QCs as references. Features with RSD > 20% were also eliminated.

### 2.5 Lipid identification

A manual inspection about putative identification was done by searching accurate mass in HMDB (https://hmdb.ca), LipidBlast (https://fiehnlab.ucdavis.edu/projects/lipidblast) and METLIN (https://metlin.scripps.edu). Putative identification was assigned based on accurate mass, retention time, and fragmentation patterns [Progenesis QI (version 3.0.7, Waters Corp., Milford, United States)]. Confidence levels in annotation were as following: level 4 (molecular formula): molecular formula identification of features is completed *via* isotope abundance distribution, charge state and adduct ion determination. Level 3 (tentative structure): tentative structural identification includes a unique match of the parent ion (MS1) data searched through literature and/or libraries and databases. Level 2 (putative identification): putative identification reveals probable structure using fragmentation data from literature and/or libraries and databases (Schrimpe-Rutledge et al., 2016). For the significant features putatively identified, a MS/MS method was performed. Briefly, precursor ions were fragmented with collision energies 10 eV, 20 eV, and 40 eV. Mass spectra were analyzed and based on the fragmentation pattern; an identification (level 2–4 of confidence) was assigned.

### 2.6 Statistical analysis

Medians with interquartile ranges (IQRs) or means [with standard deviation (s.d.)] and frequencies (%) were used to describe baseline characteristics of non-COVID-19 subjects, and COVID-19 or post-COVID-19 patients for continuous and nominal data, respectively. Normality was assessed using the D'Agostino-Pearson normality test. Continuous variables were analyzed using Mann-Whitney U or Kruskal-Wallis tests. For nominal variables (e.g., sex, smoking, death, symptoms, and comorbidities) chi-square tests for trends were used. All *p*-values less than 0.05 considered statistically significant. Analyses were conducted using GraphPad Prism version 8.0.1 for Windows (GraphPad Software, La Jolla California United States).

For lipidomics data, functional and statistical analyses were performed with MetaboAnalyst 5.0 (https://www.metaboanalyst.ca). After filtering and eliminating possible artifacts or redundancies, data was normalized by sum (TIC), transformed by square, and scaled by range.

Mummichog pathway activity profile was done with the intention to reveal the most important metabolic pathway altered without dealing with identification of all features. Mass tolerance was set to 5 ppm, *p*-value: 10^–4^ cut off (default top 10% peaks), pathway library: *homo sapiens* (human) metabolite sets for lipids (main chemical classes and sub-classes) and metabolic pathways with at least three entries were considered. Metabolite sets were manually curated and originate from a number of sources (KEGG, BiGG, and Edinburgh Model).

Analysis of continuous and categorical data was performed by Mann-Whitney rank sum and Fisher’s exact tests, respectively. Adjusted *p*-values (false discovery rate, FDR) < 0.05 were considered as significant. Univariate analysis of covariance (ANCOVA) was conducted in SPSS (version 29, SPSS Inc., United States) to examine the differences between post-COVID, COVID-19 patients and negative controls, including all features from the lipidomic dataset adjusted by age and comorbidities that were reported as significant (presence or absence of diabetes and obesity).

Principal component analysis (PCA) and two-dimensional partial least squares discriminant analysis (2D PLS-DA) scores plots were used to compare plasma lipidomic data across and between study groups; 2000-fold permutation tests were used to minimize the possibility that the observed separation of the PLS-DA was due to chance. Discriminant Q^2^ (DQ^2^) is an improvement for the Q^2^ value used in the validation of PLSDA models since it does not penalize class predictions beyond the class label value. DQ^2^ estimations were performed using Matlab (version 2020B, The MathWorks, United States) using the DQ^2^ Matlab routine written by Westerhuis et al. (Westerhuis et al., 2008) (http://www.bdagroup.nl/). Variable importance in projection (VIP) and heat maps were also plotted. Significant features were considered when having a VIP score >1.5 and a FDR <0.05.

## 3 Results

Table 1 shows the baseline characteristics of negative controls, COVID-19 patients (acute phase), and post-COVID-19 patients (2 years after recovery). At the time of the clinical examination, none of these patients had an active infection.

**FIGURE 1:**
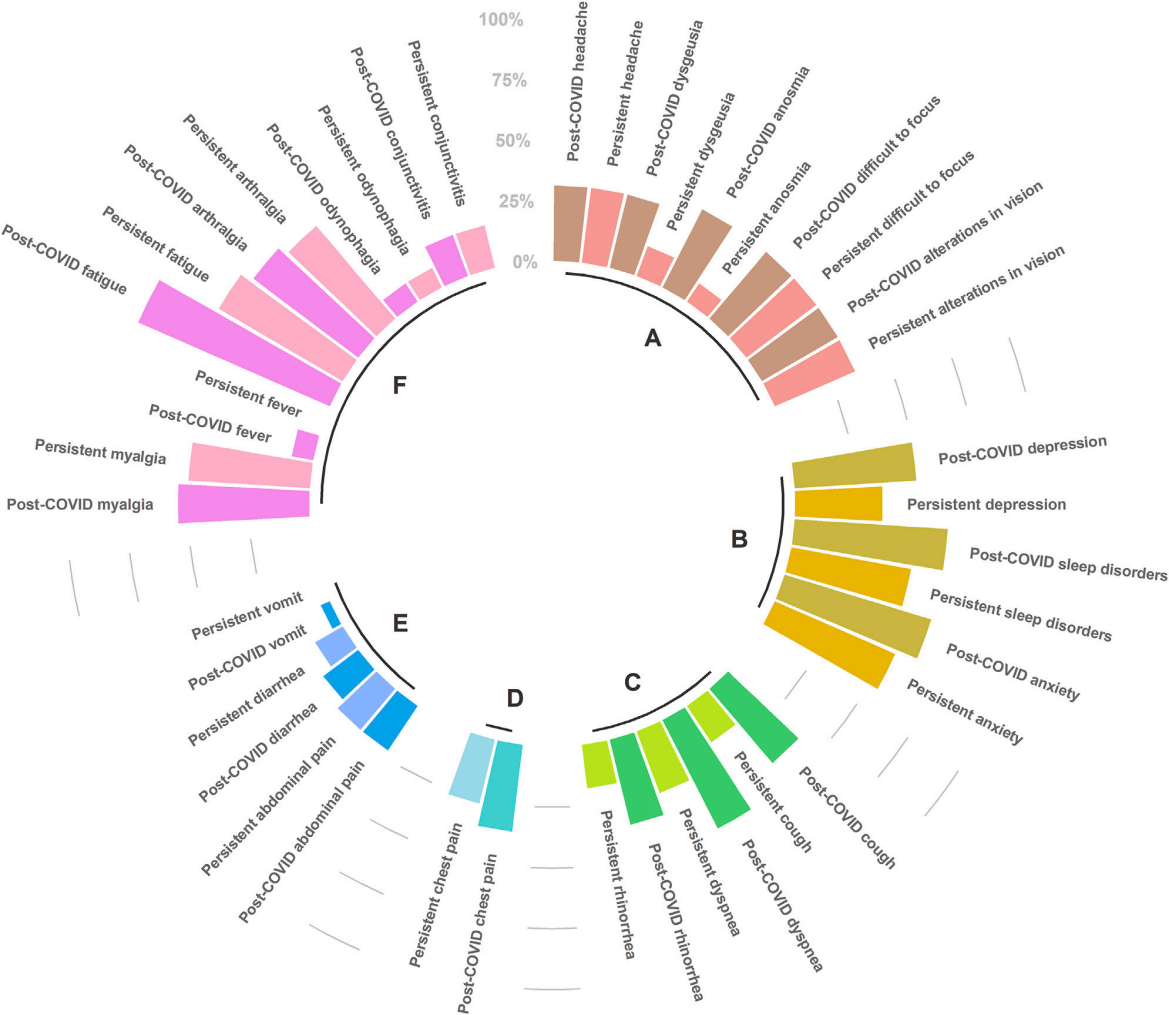
Post-COVID-19 (immediately after infection resolution), and persistent symptoms (until September 2022), clustered by etiology condition. **(A)** Neurological, **(B)** Psychiatric, **(C)** Respiratory, **(D)** Cardiac, **(E)** Digestive, **(F)** Systemic. The scale (0%–100%) represents the percentage of patients reporting each particular symptom. Figure was built with R package (ggplot2). Only symptoms that were non-significant (chi^2^ test) between post-COVID-19 and persistent status were represented.

Among the 20 patients with post-COVID-19, 55% were male and the mean age was 51.8 ± 11.6 years. Four (27.3%) patients had developed a mild disease during the acute phase, 11 (50%) had severe disease, and five (22.7%) were critically ill requiring intubation. After two years, most of the laboratory parameters were normal. Lymphocytes, monocytes and neutrophils, which were altered parameters during the acute phase, showed statistical differences with the post-COVID phase. Various clinical symptoms persisted after two years of recovery; patients basically reported the same symptoms of the acute phase, except for vomiting and fever (Figure 1).

**FIGURE 2:**
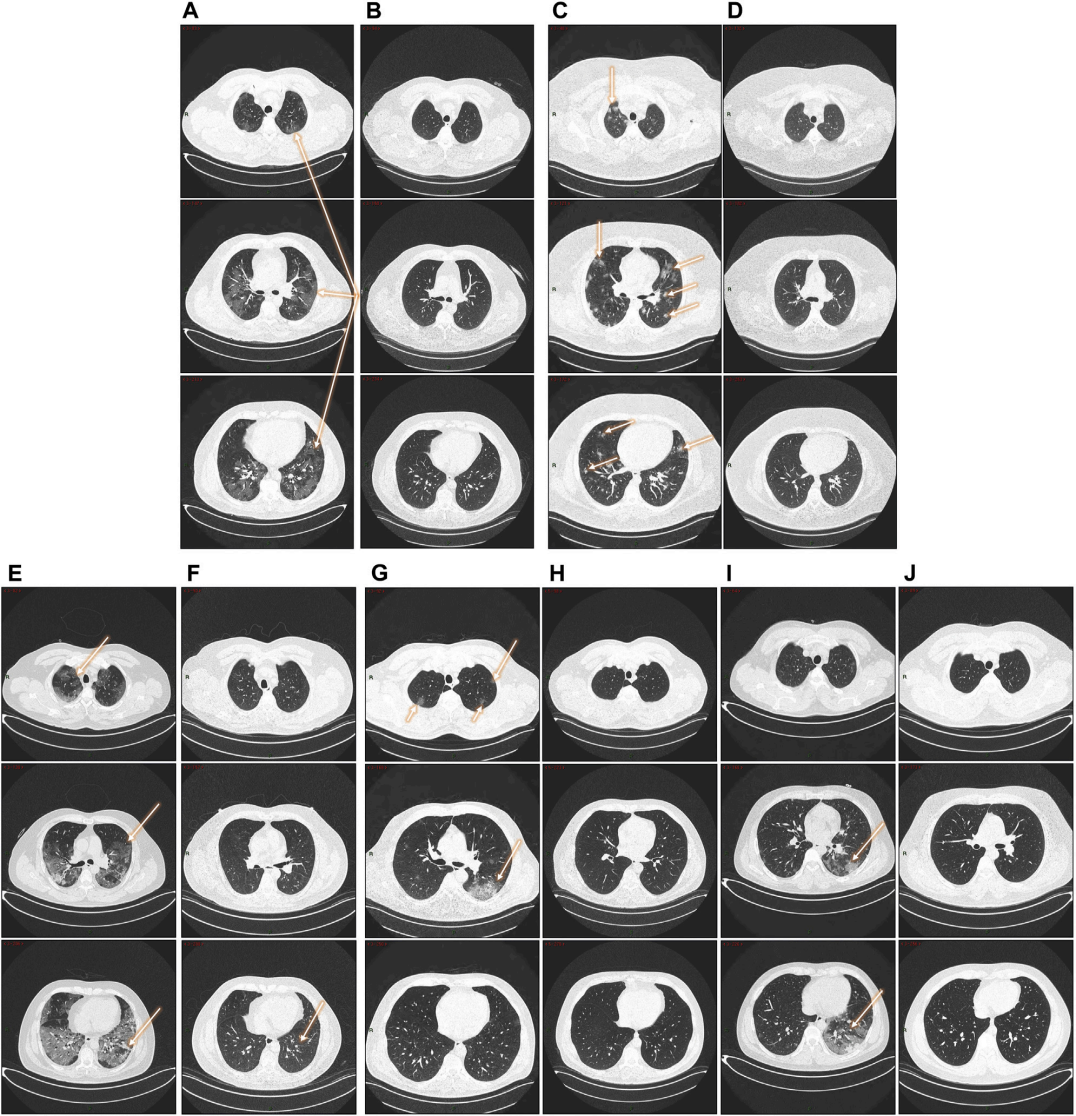
Follow-up lung CTs in patients with different degree of severity. **(A, B):** 47-year- old male patient (severe disease). **(A)** 2020. Pneumonia with presence of a ground glass pattern in the three levels of the parenchyma, with a predominant injury in the left basal region (arrows) with scattered areas of consolidation. **(B)** 2022. Complete resolution of radiological findings. **(C, D):** five- year-old male patient (severe disease). **(C)** 2020. Multiple nodular opacities in the right upper lobe (arrow) with ground glass images in the middle level of the parenchyma (coronal section) and predominance the left lobe. At the basal level, the affected areas are smaller with a diffuse nodular pattern. **(D)** Complete resolution of radiological findings. **(E, F)**: 51-year- old male patient (severe disease). **(E)** 2020. Pneumonia with the presence of a pattern in ground glass patches in the three levels of the parenchyma, with injury predominantly in the left basal region (arrows) with areas of consolidation and a pattern of bronchoalveolar distention. **(F)** 2022. Almost complete resolution of COVID-19 pneumonia with a mild residual interstitial pattern, predominantly at the basal level, coronal section (arrow). **(G, H):** 51-year-old male patient (severe disease). **(G)** 2020. Ground glass-type opacities are identified in the middle level of the parenchyma in coronal section, left lobe (arrow). At the apical region, a diffuse pattern of cotton-nodular appearance. **(H)** 2022. Complete resolution of the radiological findings. **(I, J):** 30-year-old male patient (severe disease). **(I)** 2020. Pneumonia with presence of a minimally affected pattern in the apical area with barely perceptible frosted glass smears, diffuse ground-glass patches at the mid-level of the parenchyma, with considerable involvement of left basal region (arrows). **(J)** 2022: Complete resolution of radiological findings.

Patients with an abnormal chest CT in 2020 were taken a follow-up CT scan to assess pulmonary sequelae. Representative CT images of the two COVID-19 groups in 2020 and 2022 are shown in Figure 2. While some patients had a complete resolution of abnormal findings two years after the initial infection, others had persistent lung abnormalities (interstitial thickening, ground glass opacity, and subpleural bands).

### 3.1 Functional analysis of untargeted lipidomics data generated from high-resolution mass spectrometry (HRMS)

**FIGURE 3:**
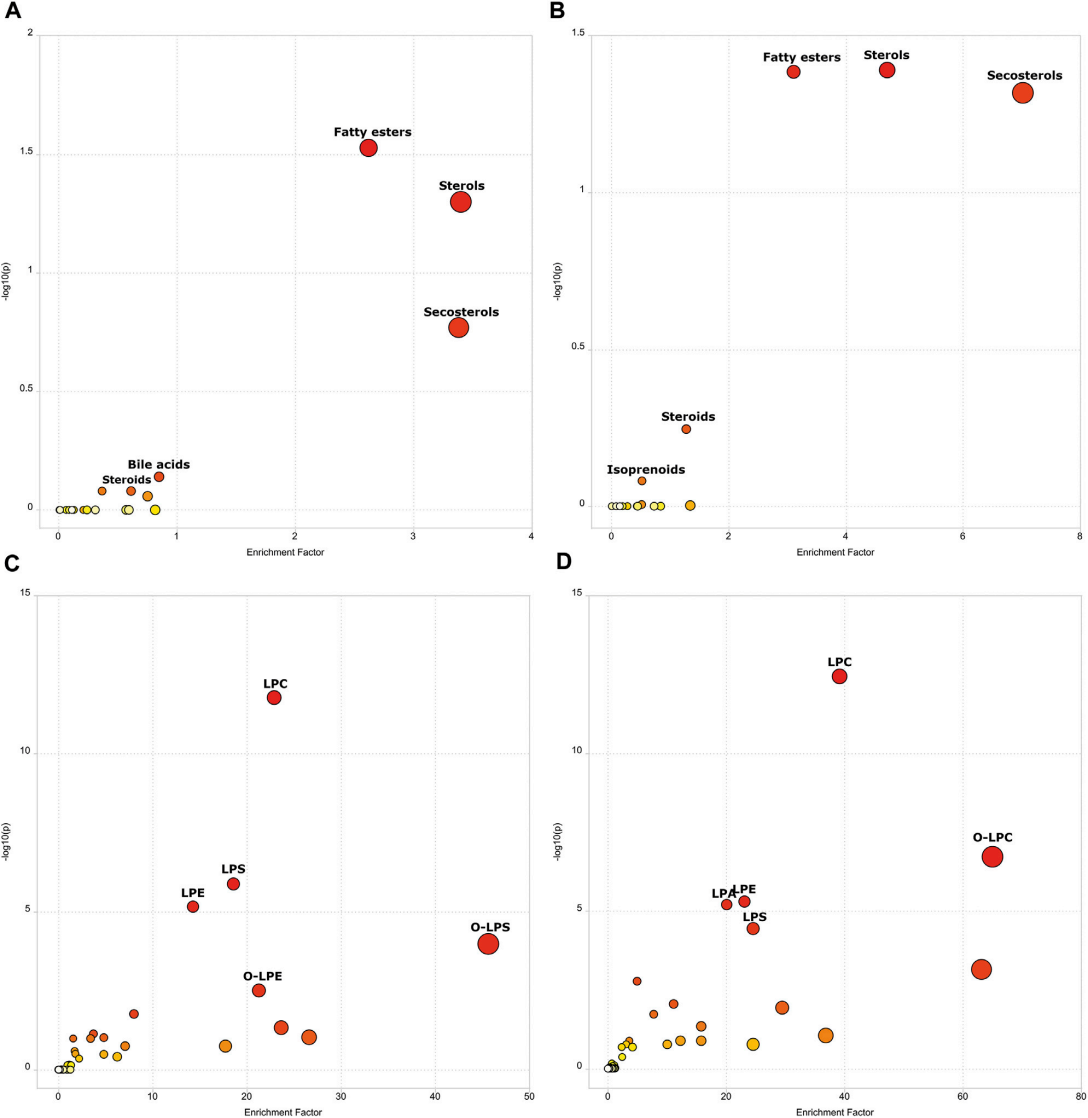
Scatter Plot representing Mummichog Pathway Activity Profile showing the enrichment factor. The enrichment factor is the ratio between the number of significant pathway hits and the expected number of hits within the pathway. **(A)** Negative controls vs COVID-19 (lipids main chemical class). **(B)** Negative controls vs Post-COVID-19 (lipids main chemical class). **(C)** Negative controls vs COVID-19 (lipids sub chemical class). **(D)** Negative controls vs Post-COVID-19 (lipids sub chemical class). Size and color increases as -log10(*p*) and enrichment factor increases. Figure was built with MetaboAnalyst software v 5.0 (https://www.metaboanalyst.ca).

With the goal to know the most important metabolic pathways dysregulated, both in the infection and recovery phases, a functional analysis (mummichog pathway activity profile) was done. A total of 401 features were detected after filtering as previously described. When the mummichog pathway activity profile was performed, putative dysregulated metabolic pathways were associated with both states (acute phase and post-COVID-19) with respect to negative controls (Figure 3). When analyzing active infection (COVID-19 group) and recovery (post-COVID-19 patients), fatty esters, sterols, secosterols and steroids were dysregulated. For active COVID-19 patients, bile acids pathway was dysregulated, and for post-COVID-19, it was the isoprenoids pathway. When subclasses of lipids were represented, both in the case of acute phase and post-COVID phase, phospholipids were the most important family of compounds dysregulated. Supplementary Tables S2, S3 show the specific details for these metabolic pathways, respectively.

### 3.2 Univariate and hierarchical clustering analysis

**FIGURE 4:**
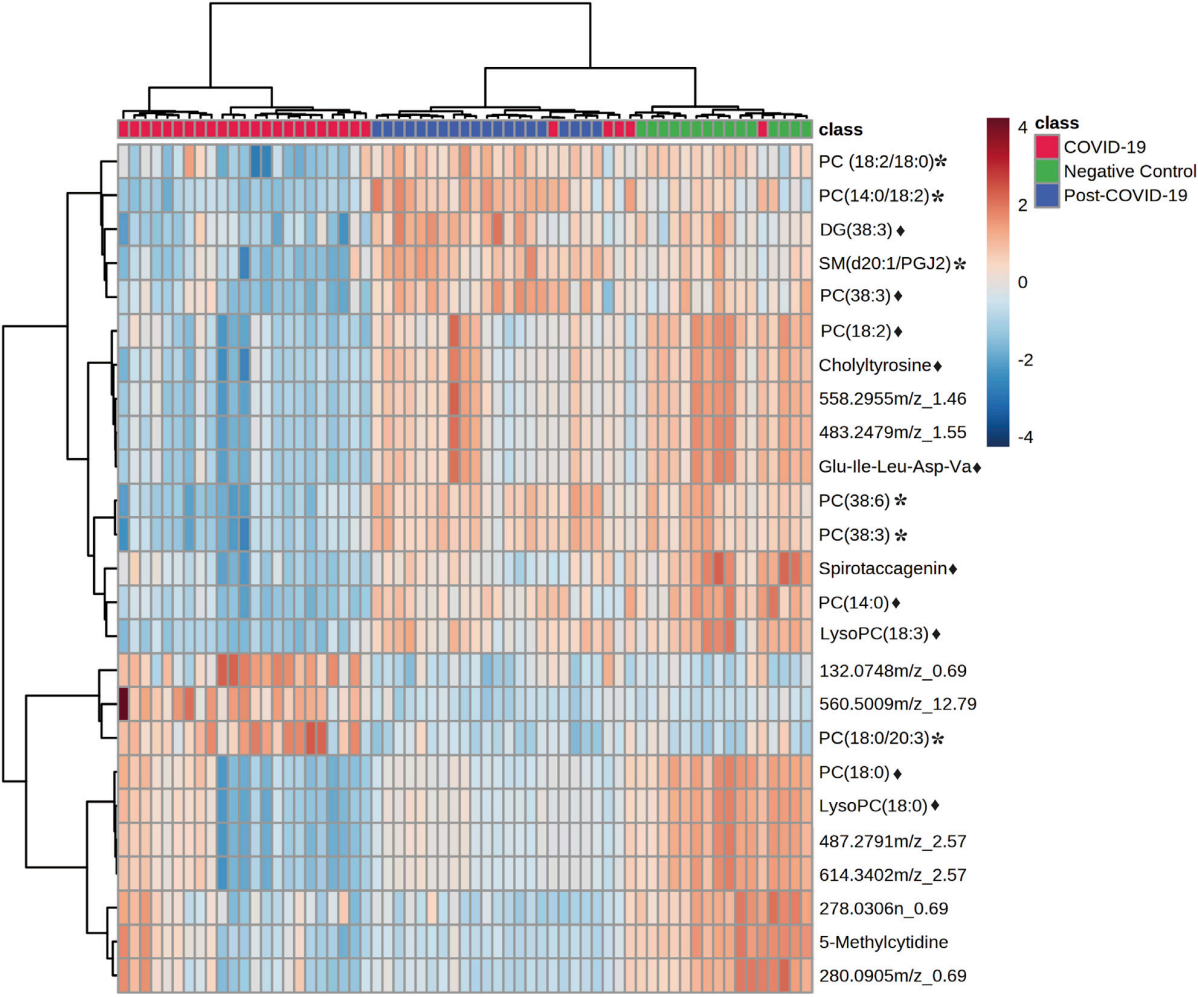
Heat map representing the most important features differentiating negative controls, post-COVID-19 patients, and COVID-19 patients. Data was normalized, distance measure: Euclidean, clustering method: Ward. Ranked by ANOVA test. Figure was built with MetaboAnalyst software v 5.0 (https://www.metaboanalyst.ca). * Represents features with confidence level 2 and ♦ represents features with level 3.

Significant statistical differences between the three study groups were observed. In total, 306 features were significantly dysregulated; 97 showed differences (FDR < 0.05) between controls and post-COVID-19, while 251 were different between post-COVID-19 and COVID-19. With respect to controls, 239 features were different compared with COVID-19 patients. ANCOVA replicated the findings of ANOVA after adjustment by age, diabetes, or obesity for the putative features (Supplementary Table S4). The heatmap revealed differences between the COVID-19 group compared with the control and post-COVID-19 groups, but there were also differences between post-COVID-19 patients and the negative controls (Figure 4).

### 3.3 Multivariate analysis

**FIGURE 5:**
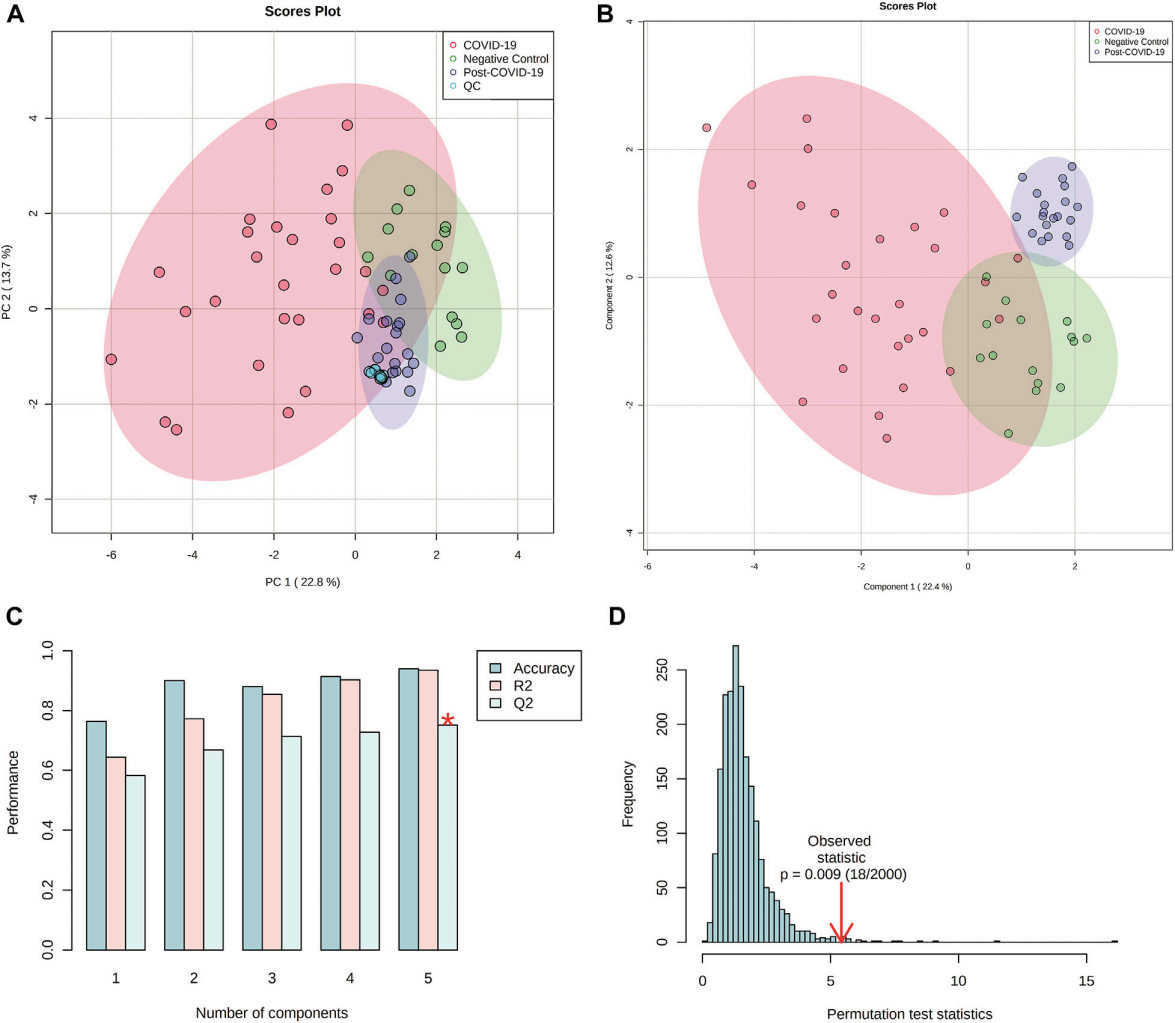
Multivariate analyses from plasma lipidomics profile of negative controls, COVID-19 patients, and post-COVID-19 patients. **(A)** Score scatter plot based on PCA models including quality controls (QCs) samples **(C)**. **(B)** Score scatter plot based on PLS-DA models. **(C)** Cross validation (10-foldCV). **(D)** permutation test (2000 permutations). Figures were produced in Metabo Analyst software v 5.0 (https://www.metaboanalyst.ca/).

The multivariate analysis showed a clear separation between the three groups. Principal Component Analysis (PCA) revealed a good clustering in QC samples indicating that technical reproducibility and stability of the system was achieved during the analysis. Once the QCs were inspected, they were eliminated from subsequent analysis. Partial Least Square Discriminant Analysis (PLS-DA) also showed good discrimination. The performance for this model was evaluated by 10-fold cross-validation and permutation test showing no overfitting (Figure 5). Double-check of the models was done as a validation resource of diagnostic statistics for PLS-DA. DQ^2^ were as follow: 0.65, 0.79 and 0.73 for the comparisons between: controls vs COVID-19, controls vs Post-COVID, and post-COVID vs COVID-19, respectively.

**FIGURE 6:**
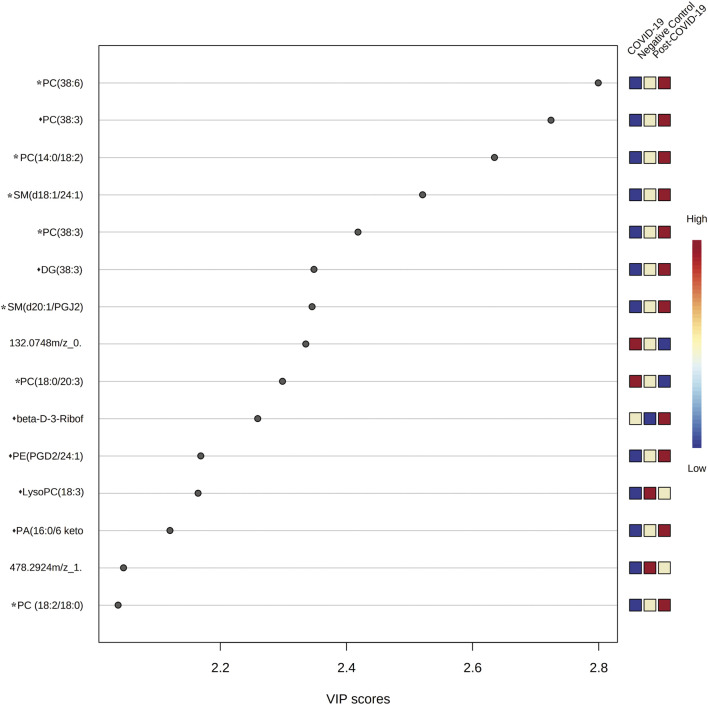
Variable importance in Projection (VIP) plot representing the rank of 15 features identified by PLS-DA according to VIP score on *x*-axis. The most discriminating metabolites are shown in descending score order. The color boxes indicate whether metabolite concentration was increased (red) or decreased (blue). Figures were produced in MetaboAnalyst software v 5.0 (https://www.metaboanalyst.ca/). * Represents features with confidence level 2 and ♦ represents features with level 3.

**FIGURE 7:**
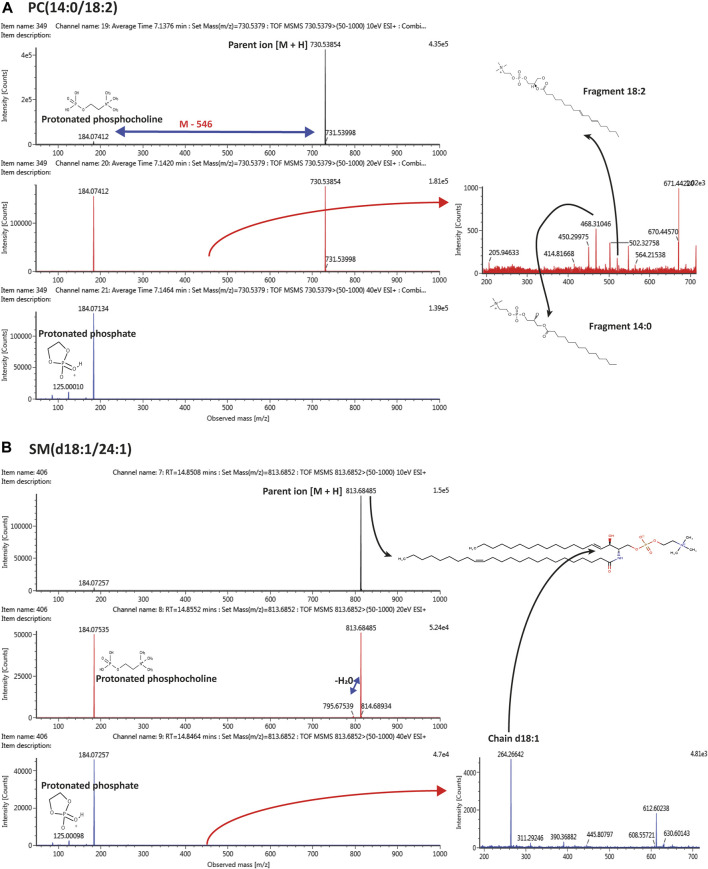
Tandem mass spectrometry (MS/MS) representing the pattern of fragmentation with 10, 20, 40 eV in ESI (+) mode, leading to the identification of **(A)** PC(14:0/18:2); **(B)** SM(d18:1/24:1).

VIP plots showed the most differentiated features with higher concentrations were found in the post-COVID-19 group (Figure 6). Details about m/z, retention time, adducts, and adjusted *p*-value are shown in Supplementary Table S4. Tandem analyses (MS/MS) were performed for the most important and abundant ions. Figure 7 and Supplementary Table S5 shows a representation of the fragmentation pattern leading to the identification of glycerophospholipids and sphingomyelins.

### 3.4 Paired analysis

Thirteen patients with paired samples (acute phase and post-COVID-19) were included; 38.4% male, and mean age 54.6 ± 7.9 years. Three (23%) developed a mild disease during the acute phase, 8 (61%) had a severe disease, and two (15%) were critically ill requiring intubation (Table 2). After two years, most laboratory parameters became normal. Lympochytes counts, altered during acute phase, was statistically different compared with the post-COVID phase. After two years of recovery, patients reported the same symptoms experienced during the acute immediate recovery phase, except for vomit and fever.

**TABLE 2:** Clinical characteristics from patients with paired samples 2 years after recovery.

Variable	COVID-19 (N = 13)	Post-COVID-19 (N = 13)	*p*-value
**Male gender, n (%)**	**5 (38.4%)**		**NA**
**Age, mean (± s.d)**	**54.6 ± 7.9**		**NA**
**Disease severity**			
**Mild, n (%)**	**3 (23%)**		**NA**
**Severe, n (%)**	**8 (61%)**		**NA**
**Critical, n (%)**	**2 (15%)**		**NA**
**Laboratory**			
**Hemoglobin (g/dL), median (Q1-Q2)**	**14.90(13.80–16.40)**	**15.40(14.50–17.40)**	**0.08**
**Platelets (×10** ^**3**^ **/mL), median (Q1-Q2)**	**238 (171.0–318.0)**	**238 (219.0–265.0)**	**0.96**
**Leukocytes (×10** ^**3**^ **), median (Q1-Q2)**	**9.2 (5.6–11.8)**	**7.3 (6.3–8.1)**	**0.36**
**Lymphocytes (%), median (Q1-Q2)**	**13.4 (7.2–22.8)**	**36.4 (31.9–38.4)**	**<0.001**
**Creatinine (mg/dL), median (Q1-Q2)**	**0.7 (0.68–0.8)**	**0.80 (0.6–0.8)**	**0.96**

Significant values (*p* < 0.05) are highlighted in bold.

Paired T-tests) showed 170 features dysregulated (FDR<0.05). The volcano plot revealed that 55 features were upregulated in post-COVID-19 patients, and 172 remained downregulated.

**FIGURE 8:**
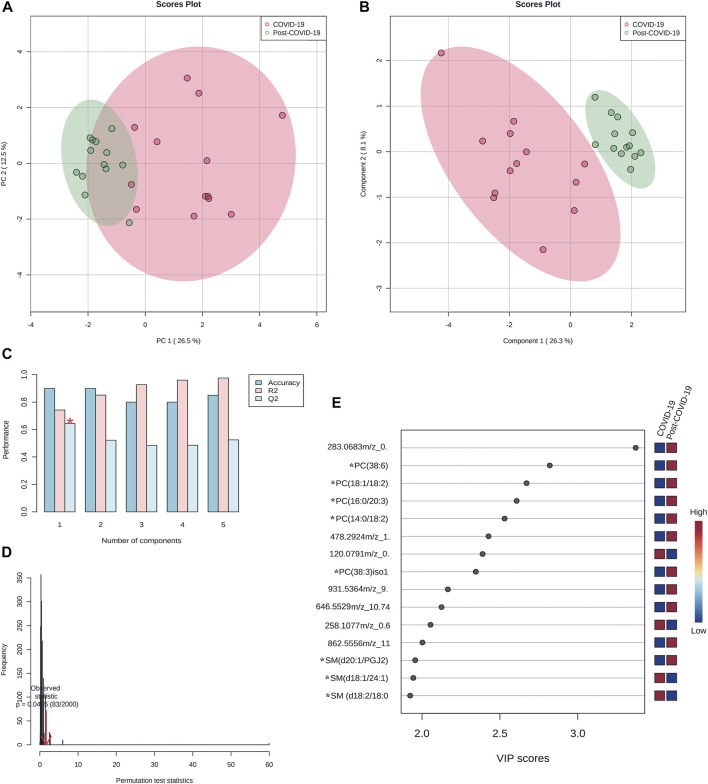
Multivariate paired analyses from plasma lipidomics profile of COVID-19, and post-COVID-19 patients. **(A)** Score scatter plot based on PCA models including paired samples. **(B)** Score scatter plot based on PLS-DA models. **(C)** Cross validation (10-foldCV). **(D)** permutation test (2000 permutations). **(E)** Variable importance in Projection (VIP) plot representing the rank of 15 features identified by PLS-DA according to VIP score on *x*-axis. The most discriminating metabolites are shown in descending score order. The color boxes indicate whether metabolite concentration was increased (red) or decreased (blue). * Represents features with confidence level 2. Figures were produced in MetaboAnalyst software v 5.0 (https://www.metaboanalyst.ca/).

Figure 8 shows the multivariate analysis. PCA (Figure 8A) revealed a good clustering of samples. Partial Least Square Discriminant Analysis (PLS-DA) also showed good discrimination; the performance for this model was evaluated by 10-fold cross-validation and permutation test showing no overfitting (Figures 8B–D).

The VIP plot showed that for most differentiated features, higher concentrations were seen among in post-COVID-19 patients (Figure 8E).

## 4 Discussion

This study aimed at describing clinical and metabolic alterations persisting two years after patients had a SARS-CoV-2 infection of different severity. The metabolic pathways dysregulated during infection and after two years of recovery were identified. A functional analysis approach was used assuming that putative annotation at individual compound level can collectively predict changes at functional levels, as demonstrated by Li et al. (Li et al., 2013).

Lipid classes belonging to sterols, steroids, and fatty esters were dysregulated in both COVID-19 groups studied. Very recently, Guntur et al. (Guntur et al., 2022) found higher levels of poly and highly unsaturated fatty acids in patients with post-COVID-19 syndrome (more than 28 days after infection: recruitment phase done in a time-interval of two years). This finding was consistent with a reduced fatty acid oxidation at mitochondrial level. The accumulation of such molecules has been associated with erythrocyte dysfunction and impairment of oxygen transportation that could persist for months, thereby explaining symptoms such as fatigue and exercise intolerance.

Sterols are a subgroup of steroids. The most familiar type is cholesterol, which is vital for the membrane structure, and it is a precursor of fat-soluble vitamins and steroid hormones. A recent systematic review and meta-analysis demonstrated that lower concentrations of total HDL, and LDL-cholesterol were significantly associated with COVID-19 severity and mortality suggesting that cholesterol concentrations might be useful for risk stratification and monitoring (Zinellu et al., 2021). Ghini et al. (Ghini et al., 2022) recently demonstrated that the lipoproteome of recovered patients slowly reverted to the healthy state.

Corticosteroids such as dexamethasone belong to steroids. They have significant anti-inflammatory and anti-fibrotic effects, which may play a role reducing lung and systemic inflammation, especially in severe pneumonia and in advanced stages of COVID-19 (Leistner et al., 2022).

Among fatty esters, monoacylglycerols, diacylglycerols, and in particular, triacylglycerols, have also been associated with metabolic dysregulation in COVID-19 patients (Masana et al., 2021).

In COVID-19 patients bile acid was also found dysregulated. Bile acids are signaling molecules with immune, metabolic, and intestinal microbiota control actions (Wahlström et al., 2016). Bile acids pathways have been widely reported in COVID-19 due to the proven association between gut dysbiosis and inflammatory processes that lead to severe disease. However, anomalies in bile acids metabolism are also associated with liver injury, and affect the substance transport system (cholesterol transport), which is common in severe COVID-19 (Shen et al., 2020). A disordered metabolism of bile acids among recovered COVID-19 patients (three months after discharge) has been documented, suggesting that the intestinal equilibrium at mucosal level is delayed before is fully repaired (Zhang et al., 2021).

In post-COVID-19 patients, the isoprenoids pathway, also recognized as mevalonate pathway (MVP) or HMG-CoA reductase pathway, was also dysregulated. Isoprenoids are a highly diverse class of biomolecules, ranging from cholesterol, vitamin K, coenzyme Q10, all steroids hormones (Holstein and Hohl, 2004). The MVP limits the activation of inflammasomes and cytokine release, and for this reason, unbalanced signaling could be associated with the pathobiology of COVID-19. A recent *in silico* study revealed dysregulation of genes involved in the MVP in SARS-CoV-2 infection, but not with H3N2 influenza virus, H1N1 influenza virus, or respiratory syncytial virus (Gomez Marti et al., 2021). Finally, the use of statins, namely, HMG-CoA-reductase inhibitors, frequently used as therapeutic agents, reduce cholesterol levels lowering viral titers through immunomodulatory, anti-inflammatory and anti-thrombotic effects (Proto et al., 2021).

These previously described metabolic alterations could account for the plethora of symptoms reported in this study. In recovered patients, values of hemoglobin, lymphocytes, monocytes, neutrophils, and creatine were within normal range two years after the acute infection. Only three out of 22 patients had one reinfection (with the Omicron variant, January 2022). By the moment of the follow-up laboratory tests, all patients tested negative for SARS-CoV-2. However, the heterogeneity of persistent symptomatology indicates that multiple organ systems were affected during the recovery phase. The etiologies of the reported conditions are post-acute COVID-19 cardiovascular syndrome, post-acute COVID-19 neuropsychiatric syndrome, and multi-system syndrome.

In the cohort of patients with baseline and follow-up CT scans, interstitial thickening, ground glass opacity, and subpleural bands were the most frequent sequelae observed. Ground glass opacity, interstitial thickening, parenchymal bands, bronchiectasis, lymphadenopathy, and pleural effusion has been reported (Yu et al., 2020). A systematic review and meta-analysis of 15 studies including over 3000 patient’s follow-up CT scans at 1–6 months after discharge showed residual CT changes in 55.7% of the cases (So et al., 2021). It could be possible that these anomalies could be reverted two years after infection in the absence of other lung diseases.

Remarkably, fatigue was the predominant alteration reported (59%), as well as musculoskeletal symptoms such as arthralgias and myalgias. Therefore, measuring the plasma lipid profile was relevant, as lipids plays an essential role in energy metabolism.

When the lipid profile was analyzed, multivariate analysis showed that after two years of recovery, post-COVID-19 patients cannot be grouped with negative controls neither clustered with COVID-19 patients, even though most basic laboratory parameters are normalized; yet, the presence of a wide spectrum of symptoms reflects that metabolic mediators are not reestablished at all.

Alterations in lipids have been found in recovered patients from SARS (2003). Wu et al. followed 25 recovered SARS patients 12 years after infection. The authors found increased levels of phosphatidylinositol and lysophosphatidylinositol (Wu et al., 2017).

In SARS-CoV-2, lipid metabolism has been reported altered in all the stages of the disease (Sun et al., 2020) and in the recovery phase. Our group has reported alteration in the levels of acylcarnitines and glycerophospholipids (phosphatidylcholines and lysophosphatidylcholines) upon admission in emergency rooms (early onset of symptoms) (López-Hernández et al., 2021). Chen and cols (Chen et al., 2022). reported that in COVID-19 patients with nucleic acid turning negative (still hospitalized), lipid metabolism was dysregulated. Acosta-Ampudia et al. also found that approximately two months after discharge, the phenotype of recovered patients did not return to a similar phenotype of pre-pandemic controls, and altered levels of unsaturated fatty acids, such as arachidonic and linoleic acid were seen (Acosta-Ampudia et al., 2021). Li et al. (Li et al., 2022) found that metabolic disturbance of lipids was associated with long-term chronic discomfort and immune dysregulation in COVID-19 survivors 6 months after discharge. The authors also reported dysregulated levels of TG, LTB4, PGE2, polyunsaturated fatty acids, including 5-hydroxyeicosatetraenoic acid (5-HETE), 12-hydroxyeicosatetraenoic acid (12-HETE), and 15-oxoeicosatetraenoic acid (15-oxoETE).

In this study, increased levels of several lipids (e.g., glycerophospholipids and sphingolipids) in the plasma of recovered patients were observed. Despite not identifying all the features dysregulated, we were able to identify (confidence level 2) the most important lipids contributing to the differentiation.

Phosphatidylcholines have been found altered in COVID-19 patients with mixed results. This is because the pattern of lipid regulation in COVID-19 patients depends upon the infection severity (asymptomatic, mild, or severe) (Hao et al., 2021). However, in most of the studies published so far, some lipid species (even within the same family) are upregulated and others downregulated, revealing a complex regulation in the context of various concomitant factors playing a role, such as the patient´s immune status and the presence of comorbidities. Here, some species of PCs were found to decrease during the active phase of the disease, and two years later these species increased in post-COVID-19 patients, even in comparison with negative controls. This could be explained as: 1) a compensatory mechanism, 2) the persistence of molecular mechanisms that are still dysregulating lipid homeostasis, and as 3) the cross-talking with the immune system and gut microbiota. In our research conducted in 2020 (Herrera-Van Oostdam et al., 2021), we observed a positive correlation between PCs and SMs with IL-12p70 and IFN-λ1. In line with this, a recent work has reported that patients with long COVID showed elevated expression of type I IFN (IFN-β) and type III IFN (IFN-λ1) that remained high after 8 months of infection (Phetsouphanh et al., 2022). On the other hand, the observed dysregulation could be due to lifestyle changes since it has been observed that mitochondrial dysfunction affect the mechanisms generating energy. Either abnormally high, or abnormally low, phospholipids can influence energy metabolism, and have large implications on general metabolic parameters (van der Veen et al., 2017).

Sphingolipids (SLs) also represent an important group of bioactive molecules involved in crucial processes such as inflammation, cellular differentiation, regeneration, aging, among others, particularly important in musculoskeletal cells (Meacci et al., 2022). The results of this study showed a dysregulation in sphingolipid metabolism, that could be associated with the reported symptoms: fatigue and muscular pain. It has been previously observed that sphingolipids impairment affect skeletal muscle cells (Danieli-Betto et al., 2005; Cowart, 2010). Accumulation of sphingolipids has been associated with inflammatory processes, and mass decrease of skeletal muscle cells of aged mice (Trayssac et al., 2018). Also, inactivity or disuse of musculoskeletal cells, as seen after disabled conditions, correlate well with remodeling of membranes enriched in SL and cholesterol along with changes in ceramide contents (Petrov et al., 2019). Ceramides and Hexocylceramides, which are derivatives of sphingomyelins, have been found increased in female patients with myalgic encephalomyelitis/chronic fatigue syndrome (ME/CFS) and among those with chronic hepatitis C infection and autoimmune disease (Zhang et al., 2016; Filippatou et al., 2021).

Increased sphingolipids levels have also been observed in metabolic syndrome (Chavez and Summers, 2012) and in the acute cell danger response (Naviaux, 2014). Regarding ME/CFS, a general agreement is that metabolic features are consistent with a hypometabolic state, characterized by a decrease in sphingolipids, glycosphingolipids, phospholipids, purines, microbiome aromatic amino acid, and branch chain amino acid. In this study, an increase in sphingolipids and phosphocholines was observed, so an underlying mechanism like ME/CFS unlikely explains the fatigue and muscular alterations seen.

Li et al. (Li et al., 2022) found that total levels of LysoPC, PA, PC, PE, PS and Cer were significantly downregulated in elderly survivors after a maximum of 9 months of a mild disease. This difference may be explained by the type of patients studied; in that study only mild disease was included, and patients were stratified by age; and the time after the acute disease was shorter.

Summarizing, our results show that post-COVID-19 is a relevant entity that requires further research.

Results also show that after two years of SARS-CoV-2 infection, some metabolic pathways are not normalized. It was worth noting that some lipid species were downregulated in post-COVID patients, while others were upregulated even within the same lipid family. These lipid dysregulations could explain some of the persistent symptoms reported by patients, especially those related to musculoskeletal disorders. Targeted studies reporting absolute concentrations for these markers are needed to establish the precise molecular mechanisms involved, and most importantly, to eventually design potential therapeutic interventions.

Finally, some important limitations ought to be acknowledged. The sample size was small due to the exploratory nature of this study; this is the result of focus given to the recruitment of patients that previously participated in protocols approved in 2020 (first epidemic wave). As a result, after two years, nearly one-third of the patients had died at hospitals within the following months after the infection. Also, from the patients that agreed to participate, there was limited data on the type and dosage of the medications prescribed during the recovery phase to be considered when interpreting the results. There was also lack of data regarding the occurrence of new illnesses and/or the reactivation of latent ones that could have affected the lipidomic profile of the patients.

All participating patients included in this study were sent to specialists to receive medical assistance for treating persistent symptoms.

## Data Availability

The dataset presented in this study can be found in Mendeley repository (www.mendeley.com) with doi: 10.17632/47n7n25z5w.1. (https://data.mendeley.com/datasets/47n7n25z5w)
