# Supplementary material for: Untargeted analysis in post-COVID-19 patients reveals dysregulated lipid pathways two years after recovery
Source: Front Mol Biosci. 2023 Mar 3;10:1100486. doi: 10.3389/fmolb.2023.1100486 (PMC10022496; doi:10.3389/fmolb.2023.1100486)
Supplement: Supplementary file 3 [file Table1.DOCX]

Supplementary Table 1: Inclusion criteria for patients (March-November 2020) and during the follow-up

| Inclusion criteria | Negative controls | Mild | Severe | Critically ill |
| --- | --- | --- | --- | --- |
| First wave (March-November 2020) | Reverse-transcriptase polymerase chain reaction (RT-qPCR) from a nasopharyngeal swab specimen negative to COVID-19 | Outpatients. Mild symptoms treated with conventional antipyretic agents. | Hospitalized patients requiring oxygen through nasal cannula. | Quick Sequential Organ Failure Assessment (qSOFA) ≥2 at the time admission, severe ARDS (PaO_2_/FIO_2_ ≤100mmHg), and reported life-threatening organ dysfunction during the illness (i.e. kidney and liver injury, and vascular and CNS complications).  Intubated patients |
| Recovered patients | Not included | Survivors willing to participate after invitation.  Absence of an active infection | Survivors willing to participate after invitation.  Absence of an active infection | Survivors willing to participate after invitation.  Absence of an active infection |
